# Characterization of morphological and chemical changes using atomic force microscopy and metabolism assays: the relationship between surface wax and skin greasiness in apple fruit

**DOI:** 10.3389/fpls.2024.1489005

**Published:** 2024-10-16

**Authors:** Yanqing Yang, Xiucui Xie, Rong Huang, Kemeng Yan, Mengdi Wang, Wenjing Liu, Xiangquan Zeng, Xiaolin Ren, Hansheng Gong

**Affiliations:** ^1^ School of Food Engineering, Yantai Key Laboratory of Nanoscience and Technology for Prepared Food, Yantai Engineering Research Center of Food Green Processing and Quality Control, Ludong University, Yantai, Shandong, China; ^2^ The Department of Spleen and Stomach Diseasess, Yantai Hospital of Traditional Chinese Medicine, Yantai, Shandong, China; ^3^ Department of Food Science, College of Agriculture, Purdue University, West Lafayette, IN, United States; ^4^ College of Horticulture, Northwest Agricultural & Forestry University, Yangling, Shaanxi, China

**Keywords:** apple fruit, skin greasiness, surface wax, chemical change, roughness parameters, atomic force microscopy

## Abstract

**Introduction:**

Skin greasiness occurred on stored apples (*Malus domestica* Borkh.) is generally believed to result from changes in surface wax components. Previous reports have typically correlated wax changes with greasiness scores to reveal the contributing wax components. A notable limitation of this approach is that greasiness scores are highly subjective and influenced by individual perception.

**Methods:**

This study aimed to assess skin quality by quantitatively analyzing wax morphology changes in greasy ‘Jonagold’ apples using Atomic Force Microscopy (AFM) roughness parameters Ra, Rq, Rmax, and Rz, and to correlate these changes with wax composition.

**Results:**

AFM results revealed that wax crystals disappeared as skin greasiness increased, accompanied by significant declines in roughness parameters Ra, Rq, Rmax, and Rz, which decreased by 70% to 85%. Chemical analysis showed a significant increase in liquid esters, including linoleate and oleate esters, in the surface waxes, which negatively correlated with the decline in roughness parameters. Key genes related to ester production, such as *MdFAD2, MdWSD1*, and *MdWBC11*, exhibited increased expression and were also negatively correlated with decreases in Rq, Ra and Rz. Additionally, 1-Methylcyclopropene (1-MCP) treatment suppressed both the development of greasiness and the associated changes.

**Discussion:**

Our findings suggest that the increased liquid esters contribute to alternations in wax morphology in greasy apples, and that *MdFAD2, MdWSD1*, and *MdWBC11*, play crucial roles in ester biosynthesis. These results highlight the effectiveness of AFM roughness parameters Ra, Rq, Rmax, and Rz in quantifying wax morphology changes in apples during skin greasiness development.

## 1 Introduction

Apples (*Malus domestica* Borkh.) are a highly favored fruit cultivated worldwide due to their high quality and long postharvest shelf life. The surfaces of apples are covered with a continuous hydrophobic wax layer, which serves as the primary barrier in preventing water loss, defending against microbial infection, and mitigating various biotic and abiotic stresses from the external environment (Chai et al., 2020; Fernández-Muñoz et al., 2022; Wu et al., 2023). The composition of apple cuticular waxes is well-documented and primarily consisting of ultra-long-chain aliphatic compounds, typically containing more than 22 carbon atoms. These compounds are characterized by their high melting points and rigid, less fluid states. The waxes include various components such as alkanes, fatty alcohols, aldehydes, and fatty acids. Furthermore, a significant amount of triterpenoids has been identified in the inner layer of the wax (Belding et al., 1998; Chai et al., 2020). The composition and content of apple waxes vary across different cultivars, developmental stages, and environmental conditions (Chai et al., 2020; Min et al., 2024).

Cuticular waxes play a significant role in determining the glossiness and appearance of fruits (Hao et al., 2024; Koch and Ensikat, 2008). Certain apple varieties, such as ‘Jonagold’, ‘Golden Delicious’,and ‘Royal Gala’, experience a solid-liquid phase transition of the wax during postharvest storage, resulting in a greasy fruit surface (Richardson-Harman et al., 2010; Yan et al., 2022; Yang et al., 2017b). This greasiness can significantly reduce consumer appeal and, consequently, the commercial value of the apples (Richardson-Harman et al., 2010). Research into the key wax components responsible for the greasiness development has evolved from focusing on changes in solid components to liquid components. Initially, attention was directed towards variations in aliphatic compounds, such as alkanes and alcohols, due to their predominant presence in cuticular waxes of apples (Curry, 2010; Dong et al., 2012; Veraverbeke and Lammertyn, 2001; Li et al., 2017). For instance, Veraverbeke and Lammertyn (2001) observed an increase in nonacosan-10-ol in cuticular waxes as skin greasiness developed in stored ‘Jonagold’ apples, suggesting that this alcohol might contribute to skin greasiness. However, nonacosan-10-ol is absent in the cuticular waxes of ‘Royal Gala’ and ‘Golden Delicious’ apples, which are still susceptible to skin greasiness development (Christeller and Roughan, 2016; Xiao et al., 2017). Recent studies have emphasized the substantial accumulation of liquid wax esters, particularly linoleate and oleate esters of (E,E)-farnesol and short-chain alcohols (C3-C5), as critical contributors to the wax changes linked to the development of greasiness in apples (Christeller and Roughan, 2016; Yang et al., 2021, 2017b). Although these liquid esters are present in low initial levels on apple surfaces, their concentration increases sharply with the development of greasiness, a trend not observed in varieties where greasiness was not apparent (Yang et al., 2017b). Thermodynamic analysis have revealed that these liquid esters may act like ‘plasticizers’, causing the solid-to-liquid phase transition of waxes as skin greasiness develops (Yang et al., 2021).

To investigate the changes in the microstructure of surface wax on apples as skin greasiness develops, Scanning Electron Microscopy (SEM) has been extensively employed. Results indicated that the wax film on the apple surface without greasiness is relatively rough, characterized by granular or plate-like wax crystals (Chai et al., 2020; Yang et al., 2017b). In contrast, during the development of greasiness, the wax crystals gradually disappear, transitioning to an amorphous state (Yang et al., 2017a, 2017b). While SEM provides objective visualization of these morphological changes, it does not quantify them. To link liquid wax components with changes in wax morphology, previous studies have commonly employed greasiness scores and wax contents for correlation analysis (Yan et al., 2018; Yang et al., 2017a, 2017b). However, the greasiness score is a subjective sensory indicator, heavily influenced by individual perception. Therefore, a more objective method is needed to accurately quantify the relationship between greasiness level and wax components.

Atomic force microscopy (AFM) is an innovative technique that provides detailed information on surface morphology by detecting interaction forces between a microcantilever probe and atoms on the sample surface, enabling precise nanometer-scale roughness measurements (Cardenas-Perez et al., 2019). AFM offers several advantages, including extremely high magnification (up to 1 billion times), versatile operational environments (such as atmosphere, vacuum, and low temperatures), minimal sample preparation requirements, and high-resolution three-dimensional imaging (Al-Rekabi et al., 2021; Misumi et al., 2015). These features make AFM highly effective for detecting material roughness (Cardenas-Perez et al., 2019). Recently, AFM has been employed in botanical research to study the nanomechanical properties of plant tissues, particularly under near-physiological conditions (Wagner et al., 2003; Zu et al., 2006). AFM has proven effective in analyzing plant leaf structures. For instance, Wagner et al. (2003) conducted a quantitative analysis of the hydrophobic structures on *Alocasia macrorrhiza* leaves, revealing numerous surface protrusions and fine structures related to cuticle folding through AFM scans. Burton and Bhushan (2006) investigated the surface structure and mechanical properties of lotus (*Nelumbo nucifera*) and taro (*Colocasia esculenta*) leaves, measuring peak-to-valley (Rmax) values of 9 μm and 5 μm, respectively. Perkins et al. (2005) utilized AFM to examine the nanoscale microstructures of *Prunus laurocerasus* leaves, uncovering surface material heterogeneity and calculating an average roughness (Ra) of 5.6 nm for rough regions and 1.4 nm for smoother areas. These studies highlight AFM’s potential for quantifying wax morphology changes in apples by assessing surface roughness as skin greasiness develops.

Apple surface greasiness often arises as the fruit overripens and is intimately connected to the ripening process. 1-Methylcyclopropene (1-MCP) is recognized for its ability to slow down the ripening of climacteric fruits by competing with ethylene for receptor sites (Li et al., 2017). Our prior research has shown that 1-MCP effectively postpones the onset of skin greasiness and mitigates changes in wax composition and structure in greasy apples. Consequently, 1-MCP serves as a valuable tool for investigating how skin greasiness influences wax modifications in stored apples.

This study aimed to assess skin quality by quantitatively analyzing wax morphology changes in greasy ‘Jonagold’ apples using AFM roughness parameters Ra, Rq, Rmax, and Rz, and to correlate these changes with wax composition. We employed AFM to measure roughness parameters and quantify changes in wax morphology as greasiness progressed. We also carefully analyzed the chemical changes in surface waxes and relative expression of genes related to wax metabolism. The responses of these changes to 1-MCP treatment were also examined. Finally, a correlation analysis was conducted to reveal the critical wax components and genes that are strongly linked to the onset of skin greasiness.

## 2 Materials and methods

### 2.1 Plant materials and treatments

Mature apple fruits of ‘Jonagold’ were harvested from an commercial orchard in Weinan city, China (35°12’25.6’’N, 109°32’49.59’’E). Apples were carefully selected to ensure uniformity in size (80 mm in diameter) and color, as well as to confirm that they were free from any physical damage. For the 1-Methylcyclopropene (1-MCP) treatment, apples were treated with 1-MCP (1 μL L^−1^) at 20°C for 24 hours (Yang et al., 2017a). The control apples underwent identical conditions for 24 hours but without 1-MCP exposure. After the treatment, the apples were placed into PVC bags, each containing around 30 apples, and then stored at 20°C with a relative humidity of 50-60% for a duration of 20 days.

### 2.2 Skin greasiness assessment

The assessment of greasiness levels in stored fruits was conducted by our previous study (Yang et al., 2021). Four levels were categorized as follows: none (0.5 > score ≥ 0), slight (1 > score ≥ 0.5), moderate (2 > score ≥ 1), or severe (score ≥ 2.0).

### 2.3 Scanning electron microscopy

Peel tissues (approximately 5 mm × 5mm) were excised from the equatorial zone of five fruits (Yang et al., 2017b). The peels were subjected to vacuum freeze-drying for 6 hours. After drying, the skin disks were coated with a gold layer using sputter coating and then examined with a scanning electron microscope (JSM-6360LV, Japan).

### 2.4 Atomic force microscopy

Following the SEM sampling method, apple peels were excised from the equatorial zone and immediately examined using an atomic force microscope (AFM; Veeco/Digital Instruments, CA) in a temperature-controlled environment (25.0°C). The AFM, equipped with top-view optics and an SNL-10 probe (Bruker AFM Probes), was operated in tapping mode with a scan rate of 0.5 to 1.0 Hz and 256 data points per line. Imaging was performed over areas of 5 μm × 5 μm and 20 μm × 20 μm. The resulting images were processed using the AFM’s proprietary software (NanoScope, Version 8.10) to reduce low-frequency noise in the slow scan direction before analysis. Roughness analysis was carried out using the same proprietary software.

Four roughness parameters were employed to assess skin quality in this study (Cardenas-Perez et al., 2019): (1) Ra (Average Roughness): Provides a general measure of the wax surface’s smoothness. A low Ra value is typically desired to ensure a fine finish and accurate reproduction of details. (2) Rq (Root Mean Square Roughness): Measures the surface roughness with a focus on larger deviations. A low Rq value indicates a more consistent and smooth finish, which is crucial for detailed mold patterns. (3) Rmax (Maximum Roughness Depth): Indicates the greatest distance between the highest peak and the lowest valley on the surface. This parameter helps in assessing any significant imperfections or irregularities that could affect the quality of the mold or casting. (4) Rz (Average Maximum Height): Provides an average of the maximum peak-to-valley heights over several sampling lengths. A low Rz value ensures that the overall texture is smooth and free from noticeable defects.

### 2.5 Surface wax extraction

Surface waxes extraction was conducted by our previous report (Yang et al., 2017b). In brief, two consecutive 45-second washes with 400 mL of distilled chloroform were applied to five apples. The combined washings were concentrated to approximately 10 mL at 40°C, dried under nitrogen flow, and stored at low temperature(-20 to -40°C) until analysis.

### 2.6 Wax preparation and chemical analysis by GC–MS

The extracts were redissolved in 20 mL mixture of methanol and chloroform (1:10, v/v), with 50 mg L^−1^ of n-heptadecane (Aladdin, China) as an internal standard (Yang et al., 2017b). Before chromatography (GC) analysis, 1 mL of each sample was dried under nitrogen and then derivatized with bis-N,N-(trimethylsilyl) trifluoroacetamide at 70°C for 1 h. (BSTFA; Aladdin, China).

Qualitative analysis was performed using a gas chromatography-mass spectrometry (QP2020NX, SHIMADZU, Japan). Compound separation was achieved with an capillary column (30 m × 0.25 mm i.d., Rtx-5 MS, 0.25 µm film), employing helium as the carrier gas (Yang et al., 2017b). A 1 μL aliquot of each sample was injected. The temperature ramping procedure was as follows: start at 70°C for 1 minute, increase to 200°C at 10°C min^-1^, then further raise to 300°C at 4°C min^-1^, and finally maintain 300°C for 15 minutes. Quantification were performed with a GC coupled with a FID detector (GC-2014, SHIMADZU, Japan). Wax compounds were identified by cross-referencing their mass spectra with the NIST17 mass spectral database, while retention indices were calculated using a series of n-alkanes ranging from C7 to C40. Semiquantitative analysis was performed by comparing the wax compounds to known quantities of the internal standard (Yang et al., 2017b).

### 2.7 RNA extraction

Peel tissues from apples, with a thickness of approximately 1.0 mm, were swiftly frozen in liquid nitrogen and stored at -80°C until analysis. Total RNA extraction and the synthesis of first-strand cDNA were carried out as described in our previous report (Yang et al., 2017a).

### 2.8 Real-time reverse transcription analysis

The expression of genes involved in the biosynthesis and transport of wax compounds was analyzed using qRT-PCR. Primers for this study were designed based on sequences as described in our previous report (Yang et al., 2017a), utilizing Premier 6.0 software. The SYBR Premix Ex Taq™ kit (TaKaRa, Japan) was employed for the reaction. The PCR program was set to 95°C for 30 s, 95°C for 10 s, 58°C for 30 s, 72°C for 20 s, 40 cycles (Yang et al., 2017a). Target gene expression was normalized to that of the internal reference gene *UBQ11* using the 2^−ΔΔCT^ method. Primer sequences are listed in **Table 1**.

**Table 1:** The primers designed for qRT-PCR.

Gene	Arabidopsis thaliana gene	Apple sequence accession no	Primer sequence (5′-3′)
*KASI*	AT5G46290	XM_008373368	AGGTGCTGGTGTATTGGTAATGG
			GCATCTTCAAGGCATCTCTGTATG
*KASII*	AF318307	XM_008380772	CAACGAGGATAGGTGGTGAGA
			CTTCTTCTGTGATTCCAGCATCT
*KASIII*	AT1G62640	XM_008365055	GTGAGAAGCGGGAAGGTGAAA
			ATTGGCAGGAGGCGAGGTT
*CAC1*	U62029	XM_008381188	CCCCAATACCCAAACAAAAACC
			GGGACAGGGAACAGTGAAGGAA
*CAC3*	AT2G38040	XM_008389895	CGCATTGCCGATAATAACACT
			TCAACGGAACACCGCTGAC
*SAD6*	AT1G43800	XM_008347059	TCCTCCCATTCCTAAAACCG
			TCTCCTCAGCCGTCCAAGAC
*FAD2*	AT3G12120	XM_008381912	TCCGTACTCTAAGCCTCCGTT
			GCACTCATGTGCTATGACCCA
*WSD1*	AT3G49200	XM_008349032	CTGGCTGATATGATGGCTAAGAAGT
			TAAGAGATTGGCAGTCAGGTATGTG
*LACS2*	AT1G49430	XM_008389114	GCTTTGAGTCGTTTCTTGTTGC
			ACTTGCTTGCCTTCGGGTT
*KCS7/2*	AT1G04220	XM_008362146	CCCTAAAAACCAACATCACCAC
			AGGCACCTCTACAGGAAACTCA
*CER4*	AT4G33790	XM_008349478	CTCTCAATACTTTGGGAGC
			AGTTTTTCTTGGAGCAGCC
*CER1*	AT1G02205	XM_017330408	CTTGGAAGCTTTAAGCATGTGG
			GGAGGAATGGTGATGTGAGTGG
*LTPG1*	AT1G27950	XM_008342003	ATTCGGTGAAGGGGATAAGG
			AGTAGTGGGAGTTGCTGTTGATG
*WBC11*	AT1G17840	XM_008360122	ATCAATGGGCATAAACAAG
			CAGAAGCAGGACCAAAATA
*UBQ11*	AT4G05050	XM_008351072	AGTCCACTCTCCATTTGGTGTT
			CCTTCCTTGTCCTGAATCTTAG

### 2.9 Statistical analyses

Each measurement was carried out in triplicate, and statistical analyses were conducted with PASW Statistics 18. Differences between different treatments were assessed using Student’s t-test. For comparisons across various storage durations, one-way analysis was employed, followed by Duncan’s multiple range test at *P*-value of 0.05. Statistical significance is indicated by * for *P*< 0.05 and ** for *P* < 0.01, highlighting differences between different treatments. Figures were created with OriginPro 9.0, while clustering analysis and correlation heatmaps were generated using R software. Data are presented as the means ± SE (n = 3).

## 3 Results

### 3.1 Assessment of skin greasiness level during storage

**Figure 1:**
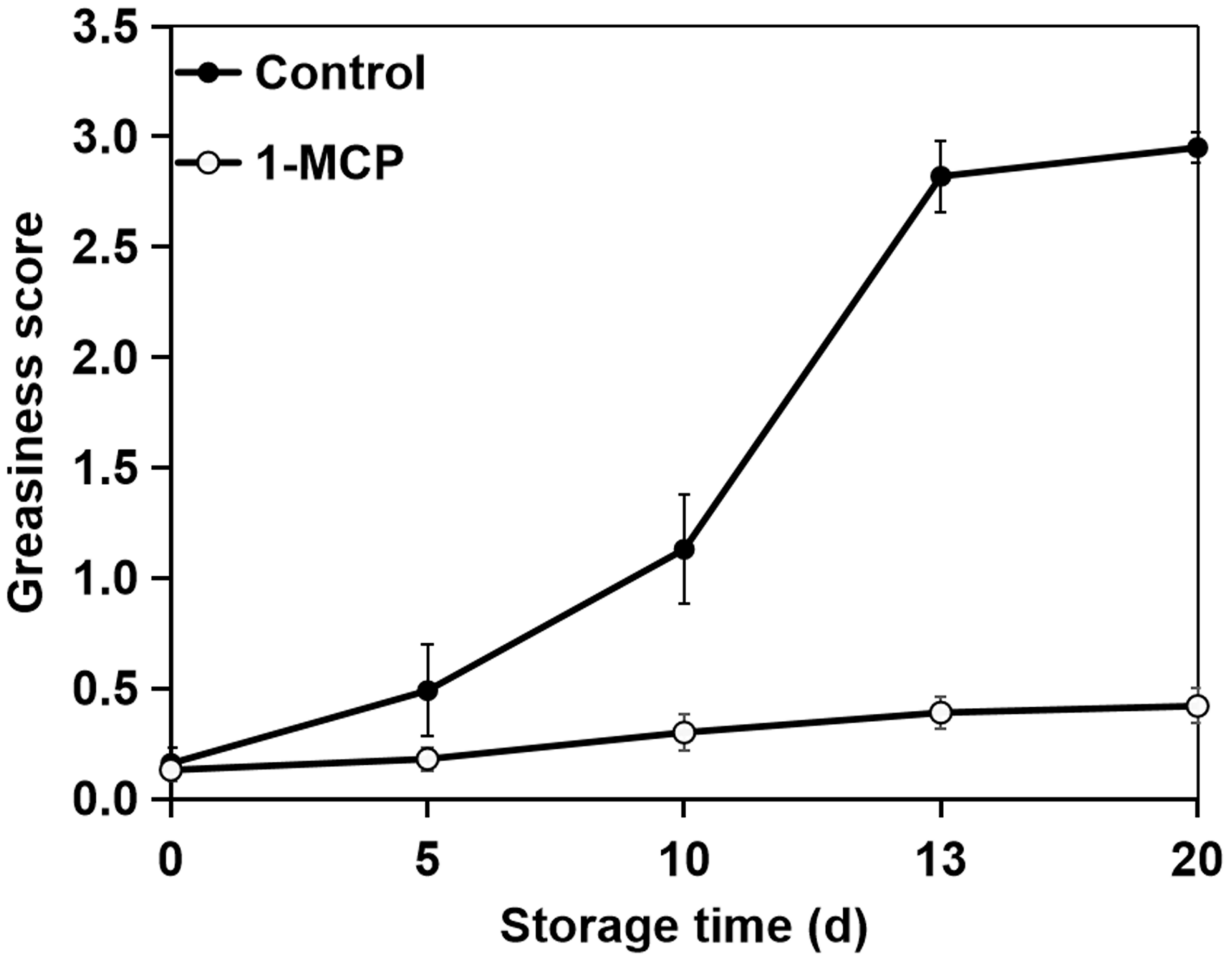
Variations in greasiness levels of surfaces on ‘Joangold’ apples during storage.

To determine the optimal sampling time, we assessed the greasiness levels of apple surfaces throughout the storage period. Following storage at 20°C, ‘Jonagold’ apples showed a gradual rise in skin greasiness (**Figure 1**). The control apples initially displayed light greasiness by day 5. This condition advanced to moderate greasiness by day 10 and escalated to severe greasiness by day 13, which remained consistently high for the rest of the storage period. Conversely, apples treated with 1-MCP did not develop greasiness throughout the entire storage period.

Apples with no greasiness (0 d), moderate greasiness (10 d) and moderate greasiness (20 d) underwent microscopy examination and chemical analysis. Apples treated with 1-MCP during the same sampling periods were also analyzed.

### 3.2 Quantitative assessment of apple wax morphology by AFM

#### 3.2.1 Comparison of morphological observations of apple surfaces using AFM and SEM

**Figure 2:**
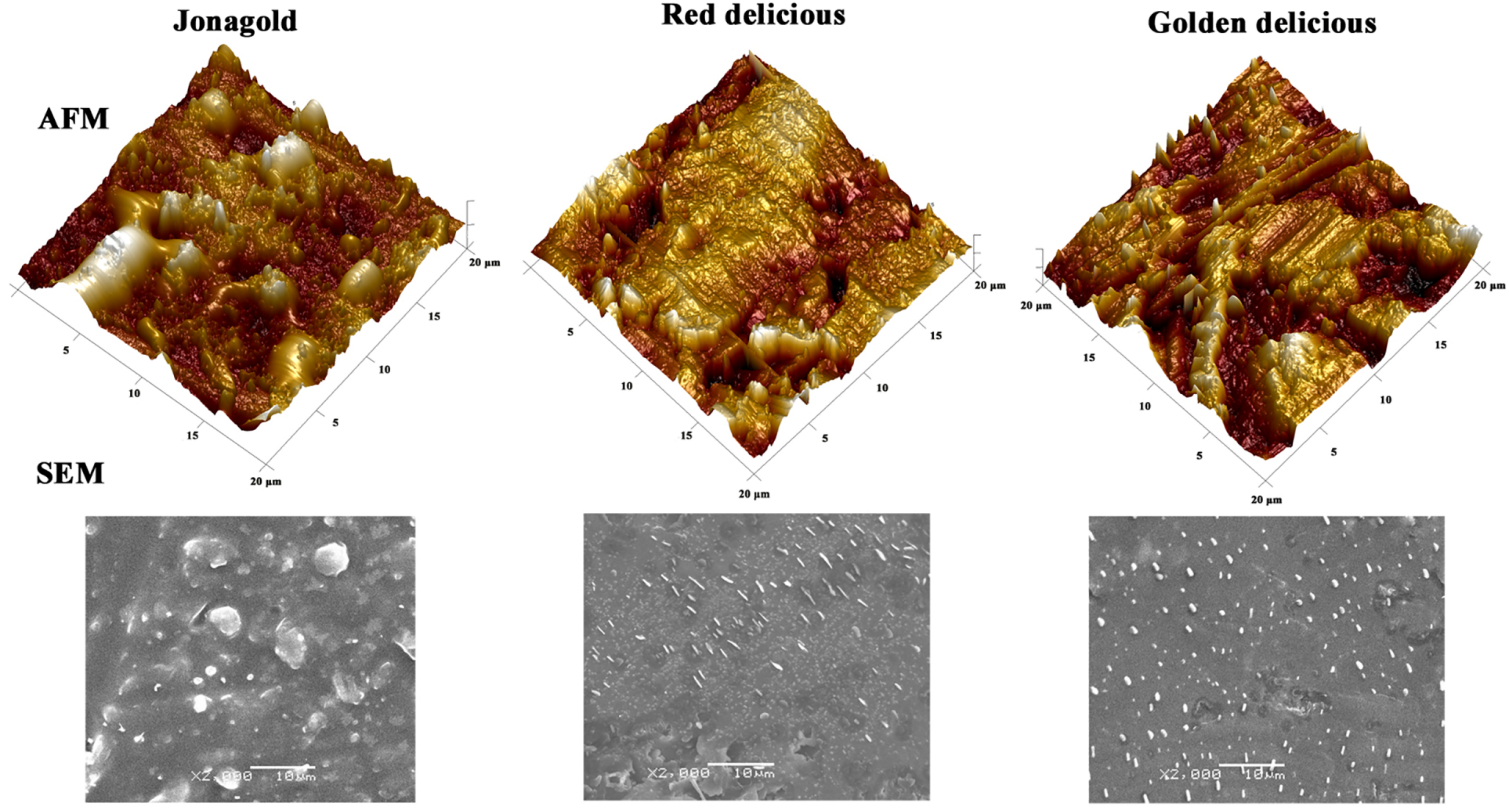
AFM and SEM images of surface waxes on mature fruit in three apple cultivars.

To illustrate the effectiveness of AFM in quantitatively assessing the morphological characteristics of fruit surface waxes, we compare morphological observations of apple surfaces using AFM and SEM. SEM images revealed a similar wax morphology across the three cultivars (‘Jonagold’, ‘Red delicious’, ‘Golden delicious’), characterized by scattered wax crystals on the surface (**Figure 2**). However, describing the specific morphology characteristics of the wax layer in words proved challenging with this method. In contrast, AFM provided three-dimensional visualizations that distinctly depicted wax morphologies unique to each cultivar. These 3D morphologies were further quantified using roughness parameters such as Rq (Root Mean Square Roughness), Ra (Average Surface Roughness), Rmax (Maximum Roughness Depth), and Rz (Average Maximum Height of the Profile). 3D morphologies (20 × 20 μm) revealed that the wax surface of ‘Golden Delicious’ apples exhibited ridges and grooves with significantly greater roughness compared to other cultivars (**Figure 2**). This difference is quantified by Ra, which represents the average surface roughness and is a standard parameter in quality control. Ra values were significantly higher in ‘Golden Delicious’ apples within scanning areas of 20 × 20 μm and 5 × 5 μm (**Table 2**). Notably, other parameters did not show significant differences in the smaller scanning area of 5 × 5 μm. Additionally, all parameter values were lower in the 5 × 5 μm scanning area compared to the 20 × 20 μm area (**Table 2**). These results suggest that scanning with a smaller area may lead to the omission of some information. Therefore, further AFM imaging was conducted in 20 × 20 μm areas to obtain a more detailed analysis.

**Table 2:** AFM-derived roughness parameters Rq, Ra, Rmax, and Rz of surface waxes in three apple cultivars.

	Rq (nm)			Ra (nm)			Rmax (nm)			Rz (nm)		
	JG	RD	GD	JG	RD	GD	JG	RD	GD	JG	RD	GD
5	51.7 ± 7.2a	50.6 ± 11.1a	83.1 ± 14.3a	23 ± 3.6a	19.1 ± 5.5a	46.35 ± 11.5b	85.0 ± 15.6a	79.3 ± 11.9a	77.0 ± 7.4a	47.5 ± 2.1a	71.4 ± 22.7b	59.5 ± 7.8ab
20	242.5 ± 29.7a	150.7 ± 15.2b	166.4 ± 23.2b	150.5 ± 32.3a	145.7 ± 13.5a	166.0 ± 14.4b	363.5 ± 38.0a	278 ± 21.4a	527.5 ± 50.2b	155.3 ± 34.5a	155.5 ± 17.8a	199.0 ± 12.8a

JG, Jonagold; RD, Red delicious; GD, Golden delicious; 5, scanning areas of 5 × 5 μm; 20, scanning areas of 20 × 20 μm; Rq, the average roughness by calculating the square root of the mean squared deviations from the surface height; Ra, the average roughness by calculating the mean of the absolute deviations from the mean height; Rmax, the maximum height difference between the highest peak and the deepest valley over a given length; Rz, the average height difference between peaks and valleys over multiple samples. Lowercase letters denote significance across three cultivars.

#### 3.2.2 Variations in roughness parameters Rq, Ra, Rmax, and Rz of surface waxes during skin greasiness development

**Figure 3:**
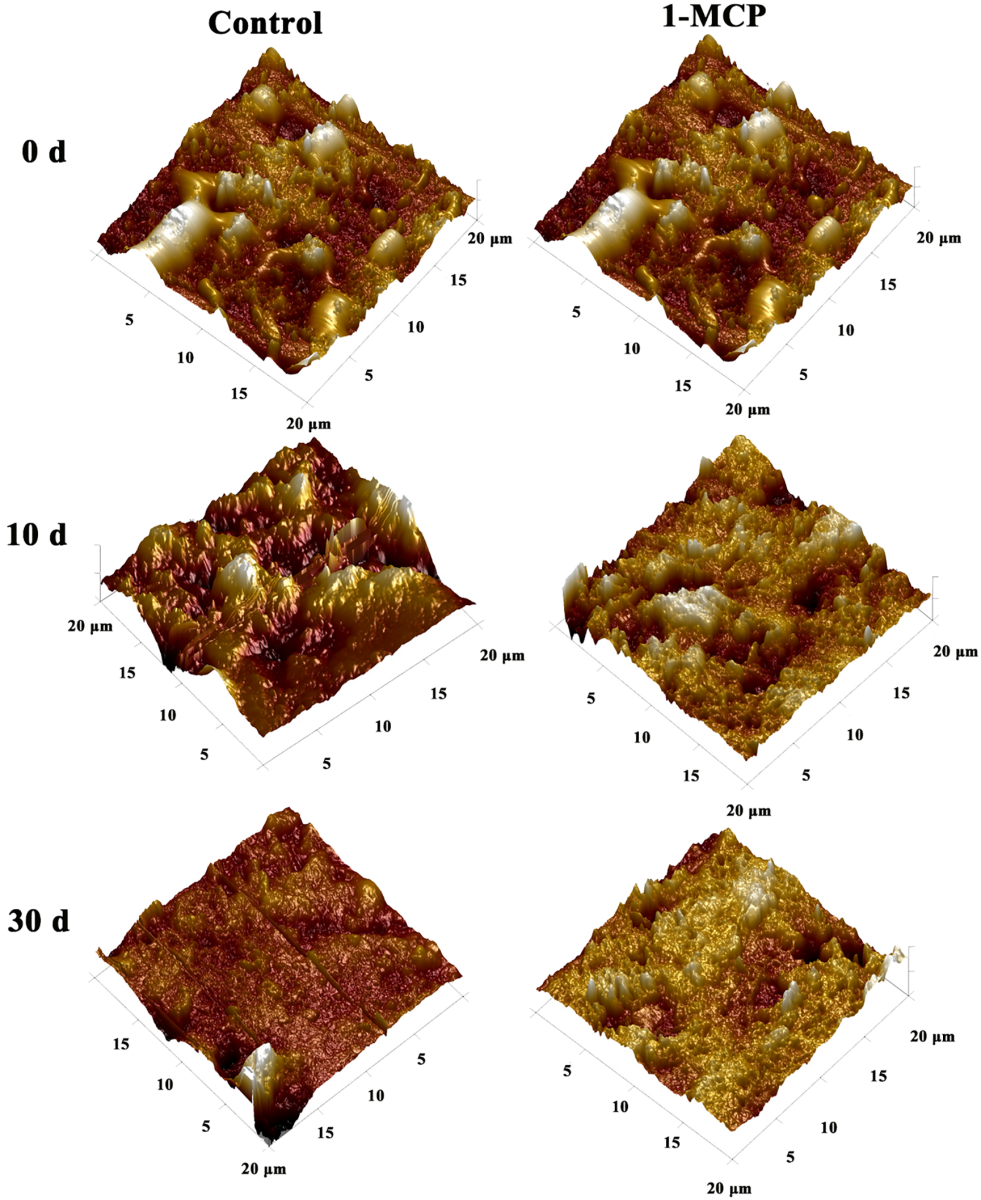
AFM-derived 3D graphics of surface waxes of ‘Joangold’ apples during storage.

The AFM was used to quantitatively analyze the morphological changes of surface waxes as skin greasiness developed in ‘Jonagold’ (**Figure 3**). Initially, on day 0, AFM-derived 3D images revealed numerous wax crystals scattered across the wax film. However, as greasiness levels increased in the control apples, there was a noticeable reduction in these wax crystals. By the end of the storage period (day 20), the wax film had lost nearly all wax crystals and displayed a glossy appearance (**Figure 3**). Concurrently, roughness parameters Rq, Ra, Rmax, and Rz of the wax film continuously decreased with increasing greasiness levels, showing a sharp decline of approximately 70% to 85% from day 0 to day 20 (**Table 3**). For example, the roughness average Ra sharply decreased from 242.5 (nm) on day 0 to 44.0 (nm) on day 20 (nm). Additionally, the results also indicated that the decrease in Rmax and Rz, which represent the maximum distance from peak to valley, corresponded with the significant disappearance of wax crystals.

**Table 3:** Roughness parameters Rq, Ra, Rmax, and Rz of surface waxes in control and 1-MCP treated ‘Jonagold’ apples stored at 20°C.

Storage time	Rq (nm)		Ra (nm)		Rmax (nm)		Rz (nm)	
(d)	CK	1-MCP	CK	1-MCP	CK	1-MCP	CK	1-MCP
0	242.5 ± 29.7Aa	242.5 ± 29.7ABa	150.5 ± 32.3Aa	150.5 ± 32.3ABa	363.5 ± 38.0Aa	363.5 ± 38.0Aa	155.3 ± 34.5Aa	155.3 ± 34.5Aa
10	74.2 ± 10.9Ba	231.9 ± 53.6BCb	48.98 ± 7.9Ba	117.6 ± 15.3BCb	259.1 ± 29.1Ba	291.2 ± 15.9Bb	100.6 ± 1.2Ba	152.9 ± 10.5Ab
20	44.0 ± 1.1Ca	196.6 ± 1.4Cb	22.6 ± 1.8Ca	97.2 ± 9.8Cb	94.0 ± 12.2Ca	286.7 ± 39.4Bb	45.3 ± 2.5Ca	154.3 ± 20.9Ab

Uppercase letters denote significance across different storage times. Lowercase letters indicate significance between the two groups.

In contrast, apples treated with 1-MCP exhibited a noticeable delay in the disappearance of wax crystals and a slower decline in roughness parameters compared to control fruit (**Figure 3**; **Table 3**). Wax crystals remained observable from day 0 to day 20 in the 1-MCP treated fruit. Importantly, roughness indices Ra, Rq, Rmax, and Rz in 1-MCP treated fruit were consistently higher than in control fruit at each time point after storage. Specifically, on day 20, roughness indices in the 1-MCP treated group were significantly higher than those in the control group on day 10, indicating preserved wax crystal structure and higher surface roughness due to 1-MCP treatment.

### 3.3 Variations in chemical composition of surface waxes during skin greasiness development

A total of 27 aliphatic compounds were identified in surface waxes throughout the storage period (**Supplementary Table S1**), including eight alkanes, five alcohols, five fatty acids, seven esters, and two sesquiterpenoids. The wax compounds were mainly composed of typical very-long-chain n-alkanes (C21-C30), followed by alcohols (C24-C30). These components accounted for approximately 40-50% and 20-30% of the total wax content, respectively, throughout the storage period (**Table 4**). Notably, all compounds classified as alkanes and alcohols were solid, while most compounds in the esters, free fatty acids, and sesquiterpenoids categories, which keep low amount at harvest, were in liquid form (**Supplementary Table S1**).

**Table 4:** Variations in wax fraction concentrations of ‘Jonagold’ apples stored at 20°C.

Storage time (d)	Wax fractions (mg m^-2^)									
	Alkanes		Fatty alcohols		Esters		Free fatty acids		Sesquiterpenoids	
	Control	1-MCP	Control	1-MCP	Control	1-MCP	Control	1-MCP	Control	1-MCP
0	4455 ± 613Aa	4455 ± 613Aa	2832 ± 252Aa	2832 ± 252Aa	346 ± 71Aa	346 ± 71Aa	383 ± 39Aa	383 ± 39Aa	215 ± 41Aa	215 ± 41Aa
10	3594 ± 150Ba	4535 ± 271Ab	2668 ± 210Aa	2587 ± 518Aa	1197 ± 84Ba	509 ± 29Bb	507 ± 54Ba	288 ± 28Bb	372 ± 8Ba	192 ± 14Bb
20	4442 ± 317Aa	4196 ± 308Aa	2561 ± 317Aa	2645 ± 210Aa	1742 ± 198Ca	536 ± 42Bb	751 ± 12Ca	368 ± 27Ab	885 ± 131Ca	232 ± 5Ab
Changes	-13	259	-271	-187	1391**	181*	368**	-15	670**	17

In addition to aliphatic compounds, triterpenic acids, which are typically embedded within the cuticle layer of a plant's epidermis, were also identified in this study. However, since skin greasiness develops on the cuticle surface, further investigation into the chemical alterations of triterpenic acids was not pursued. Uppercase letters denote significance across different storage times. Lowercase letters indicate significance between the two groups.

**Figure 4:**
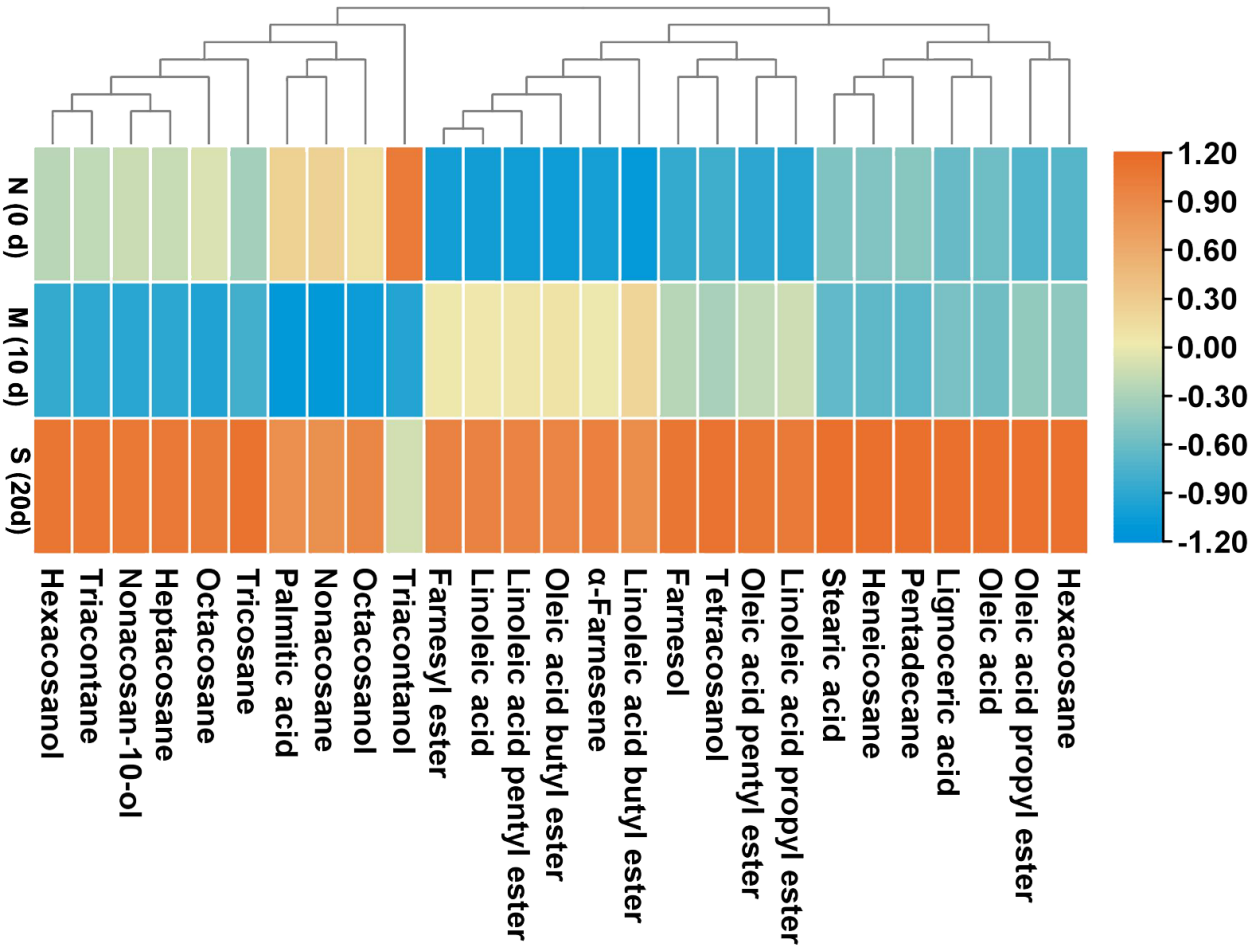
Heatmap and clustering of the surface wax compounds in ‘Joangold’ apples during storage. N, none greasiness; M, moderate greasiness; S, severe greasiness.

**Figure 5:**
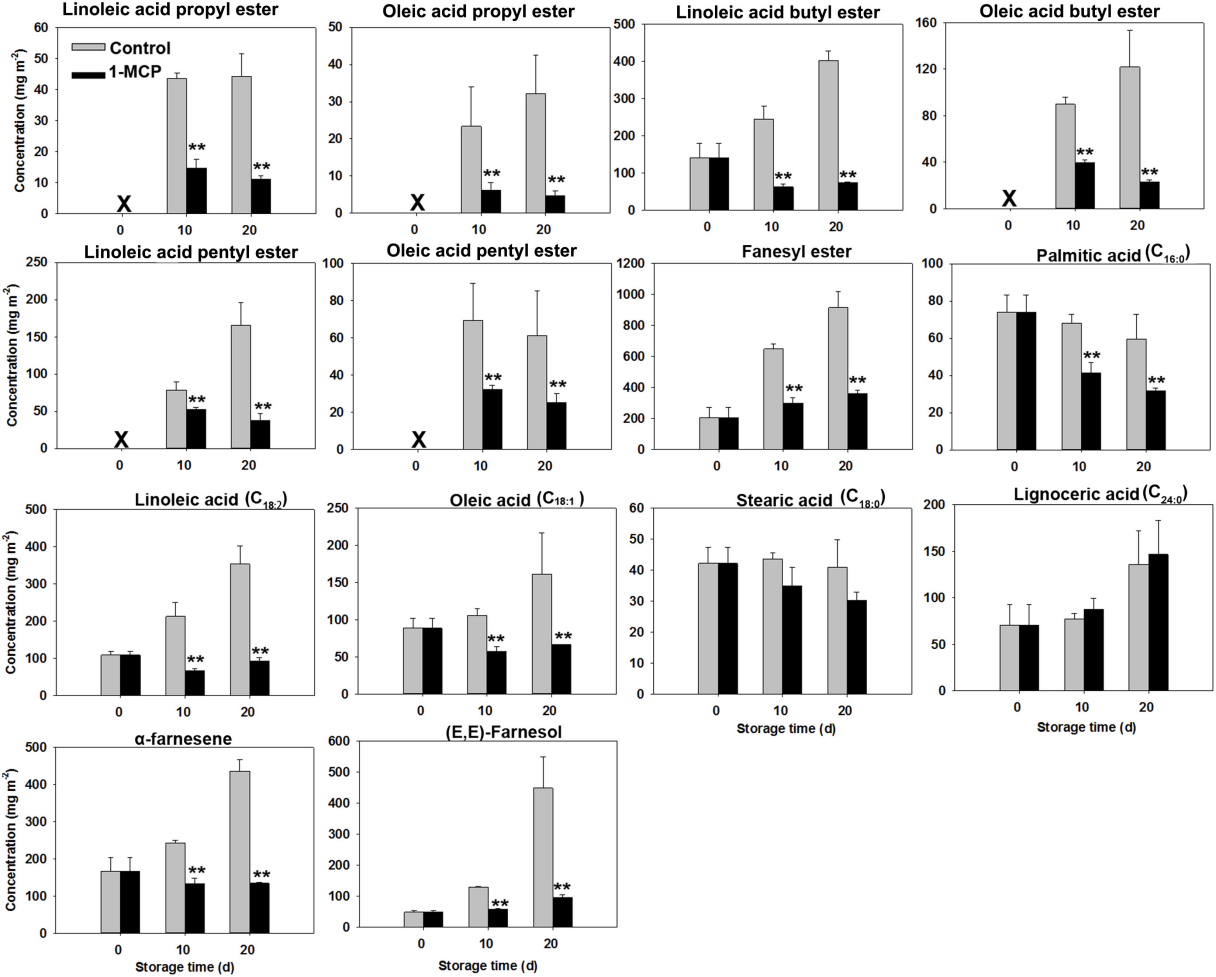
Variations in liquid wax compound concentrations in ‘Joangold’ apples during storage. X - undetected. Farnesyl ester, including farnesyl linoleate and farnesyl oleate, was considered a single compound in this study. ** represent significance (CK vs 1-MCP) at *P* ≤ 0.01.

At harvest, the liquid wax fractions, including esters, fatty acids, and sesquiterpenoids were minimal, comprising approximately 4%, 4%, and 2% of the overall wax composition, respectively (**Table 4**). However, their levels steadily increased as skin greasiness progressed in control fruit during storage. Cluster analysis clearly distinguished between liquid and solid wax components (**Figure 4**). In control fruit, ester concentrations consistently increased from an initial 351 mg m^-2^ to 1742 mg m^-2^ over the storage period, accounting for nearly 60% of the total wax accumulation. (**Table 4**). Seven esters including linoleic acid propyl ester, oleic acid propyl ester, linoleic acid butyl ester, oleic acid butyl ester, linoleic acid pentyl ester, oleic acid pentyl ester and farnesyl ester were detected (**Figure 5**). Farnesyl ester (linoleic acid farnesyl ester and oleic acid farnesyl ester was considered a single compound in this study) was the most abundant, contributing more than 50% of the total increase in esters, followed by linoleic acid butyl ester at 18%. Throughout the storage period, linoleate esters predominated over oleate esters (**Figure 5**). Conversely, apples treated with 1-MCP exhibited significantly lower ester concentrations compared to the control fruit at each time point during storage (**Table 4**; **Figure 5**). For instance, ester concentrations in treated apples (536 mg m^-2^) were approximately 33% of those in control apples (1742 mg m^-2^). Alongside the esters, the analysis also identified five free fatty acids. Over time, linoleic acid (C18:2) and oleic acid (C18:1) showed a marked increase in their prominence (**Figure 5**). The C18:2 accounting for 66% of the total rise in fatty acids. This higher content of C18:2 aligns with its predominance in esters.

**Figure 6:**
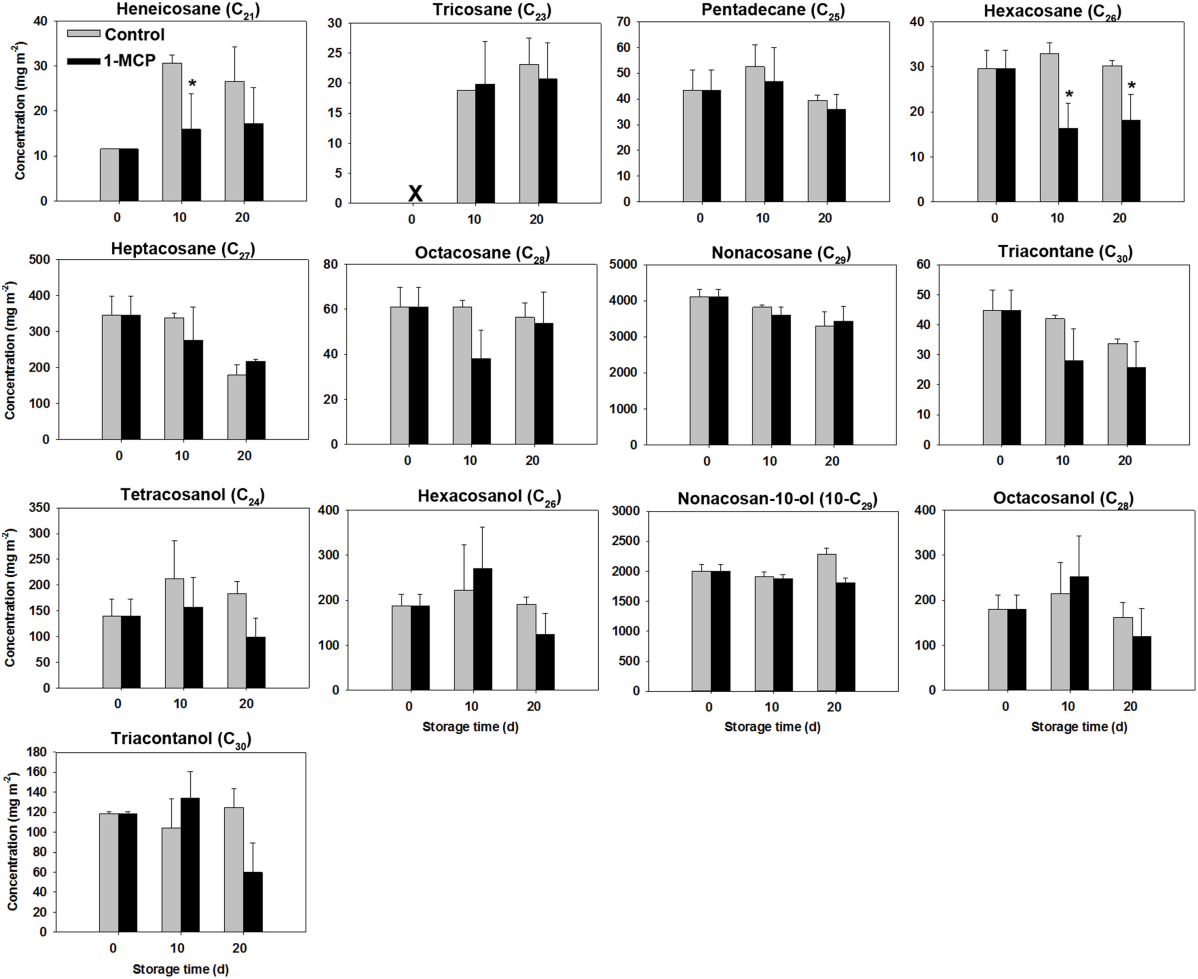
Variations in solid wax compounds concentrations in ‘Joangold’ apples during storage. X - undetected. * represent significance (CK vs 1-MCP) at *P* ≤ 0.05.

Unlike the substantial increase observed in liquid waxes, the levels of most solid wax compounds stayed relatively stable throughout the storage period for in both two groups (**Figure 6**).

### 3.4 Variations in expression of key genes related to wax formation during skin greasiness development

**Figure 7:**
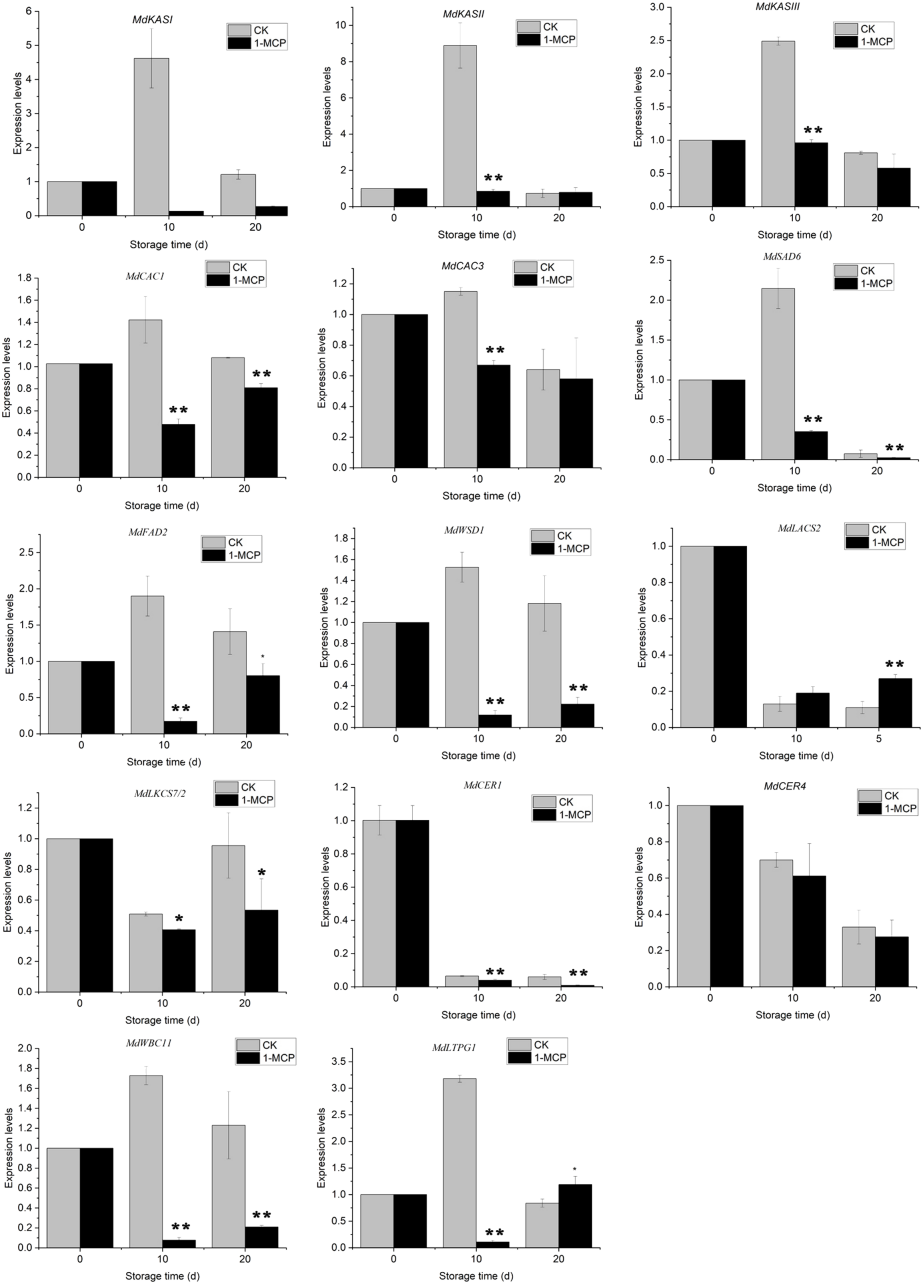
Variations in the relative expression of wax biosynthesis and export genes in ‘Joangold’ apples during storage. Transcript levels of each gene were normalized to the initial time (day 0), which was designated as 1. * and ** represent significance (CK vs 1-MCP) at *P* ≤ 0.05 and *P* ≤ 0.01.

In addition to analyzing the chemical composition of wax constituents, we investigated several genes associated with wax metabolism (**Figure 7**). The expression levels of key genes involved in *de novo* synthesis (*MdKASI*, *MdKASII*, *MdKASIII*), desaturation (*MdFAD2* and *MdSAD6*), and esterification (*MdWSD1*) of fatty acids increased in control fruit, with a notable rise on day 10. Specifically, *MdFAD2* and *MdWSD1*, which are essential for the biosynthesis of C18:2 acid and its esters, exhibited consistently elevated expression levels on both day 10 and day 20. This upregulation correlates with the increased predominance of linoleate esters in the liquid waxes. In contrast, apples treated with 1-MCP did not show upregulation of these genes after storage and had lower expression levels compared to the control fruit.

Moreover, after storage, the expression of genes responsible for synthesizing very long-chain wax compounds - namely *MdLACS2*, MdKCS7/2, *MdCER4*, and *MdCER1* - fell below the levels observed at harvest in both control and 1-MCP-treated apples, indicating that 1-MCP treatment had minimal impact on these genes’ expression (**Figure 7**). Concurrently, the expression of lipid transport genes *MdWBC11* and *MdLTPG1*, which are associated with liquid wax synthesis, increased on day 10 but was suppressed by 1-MCP treatment.

### 3.5 Correlation analysis of roughness parameters, wax components, and related synthesis genes

**Figure 8:**
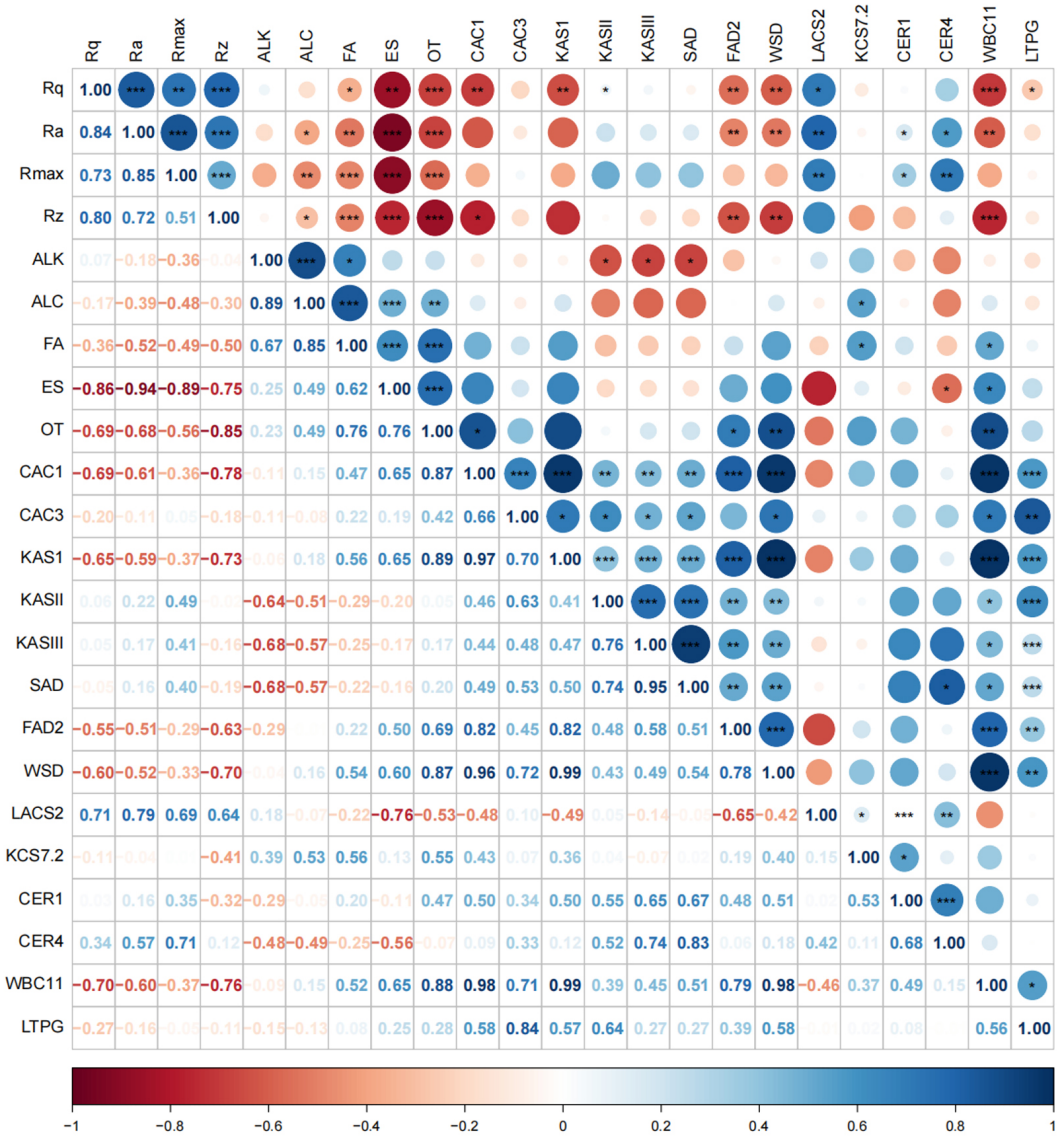
Correlation analysis of roughness parameters, wax contents and gene expression. ALK, alkanes; ALC, alcohols; FA, fatty acids; ES, esters; OT, sesquiterpenoids.

**Figure 9:**
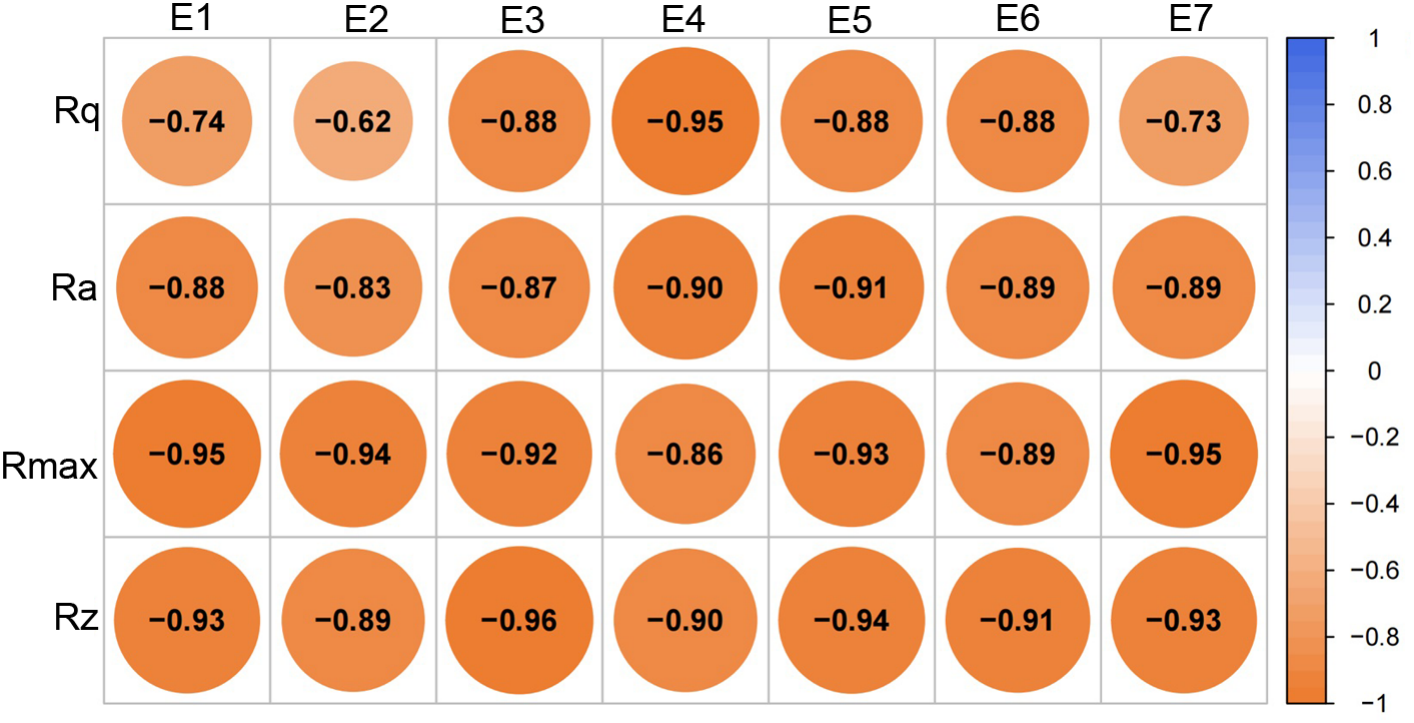
Correlation analysis of roughness parameters and ester contents. E1 - linoleic acid propyl ester, E2 - oleic acid propyl ester, E3 - linoleic acid butyl ester, E4 - oleic acid butyl ester, E5- linoleic acid pentyl ester, E6 - oleic acid pentyl ester, E7 - farnesyl ester.

To further elucidate the key wax compositions contributing to changes in wax morphology during the development of skin greasiness, correlation analyses were conducted between roughness parameters (Rq, Ra, Rmax, and Rz) and wax components along with related synthesis genes (**Figure 8**). The content of esters, fatty acids and exhibited significant negative correlations with changes in Rq, Ra, Rmax, and Rz. Specifically, all ester components showed a strong negative correlation with these roughness parameters (**Figure 9**). This suggests that an increase in liquid wax components contributes to a reduction in wax film roughness. Conversely, alkanes, despite being the most abundant wax fractions, showed no significant relationship with morphological changes as skin greasiness developed, likely due to their minimal variability. Additionally, the analysis revealed significant negative correlations between key genes involved in ester synthesis and transition, such as *MdFAD2*, *MdWSD1* and *MdWBC11* roughness parameters Rq, Ra and Rz. This underscores the critical role of *MdFAD2*, *MdWSD1* and *MdWBC11* in influencing wax morphological changes as skin greasiness progresses.

## 4 Discussion

### 4.1 The significant rise in liquid waxes was the key feature of surface wax changes in apple fruit during skin greasiness development

Apple surface waxes primarily consist of ultra-long-chain (C > 22) aliphatic compounds, which exist in a solid state and form wax crystals, resulting in a rough wax film (Belding et al., 1998; Chai et al., 2020; Curry, 2010; Liu et al., 2015), as confirmed by this study. However, when apple skin greasiness develops, the solid wax surface becomes greasy, causing an unpleasant tactile experience. Previous research on this phenomenon has focused on the changes in these aliphatic compounds (Belding et al., 1998; Dong et al., 2012; Veraverbeke and Lammertyn, 2001). Most studies indicate that these solid wax compounds undergo minimal alteration during treatment and sampling periods. Although some minor constituents show variability, such differences are insufficient to explain the subtle changes in surface wax morphology (Curry, 2010). Notably, it has been observed that when skin greasiness occurs on apples, the wax transitions from a solid to a liquid phase (Yang et al., 2017b). This finding suggests that previously unidentified liquid wax components may play a critical role in the development of skin greasiness.

Recently researches have identified a significant rise in novel liquid wax esters as a major change associated with the development of skin greasiness in apples various varieties and treatments (Lee et al., 2024; Yang et al., 2021, 2017b). The levels of these esters are highly correlated with the greasiness scores (Lee et al., 2024; Yang et al., 2021, 2017b), as confirmed by this study. Typically, plant wax esters are solid, long-chain fatty acid esters of long-chain fatty alcohols (Barney et al., 2015; Kunst and Samuels, 2003). However, in greasy apples, the identified liquid esters are oleate and linoleate esters of short-chain alcohols (C3-C5) (Yang et al., 2021, 2017b). Specifically, eight esters have been reported, including linoleate and oleate esters of (E,E)-farnesol, propanol (C3), butanol (C4), and pentanol (C5) (Yang et al., 2021, 2017b), all of which were also detected in this study. These esters are present in low amounts in apples at harvest, and their increase is more pronounced in greasiness-susceptible cultivars compared to those that are less susceptible (Yang et al., 2017b). Furthermore, our findings indicate that 1-MCP treatment inhibited the formation of these esters while delaying the development of greasiness and changes in wax morphology. This further underscores the crucial role of liquid wax esters contribute to alternations in wax morphology in greasy apples.

### 4.2 Quantifying skin greasiness development via roughness parameters (Rq, Ra, Rmax and Rz) contributes to identifying key associated wax compounds

As skin greasiness progresses, surface waxes transition from a solid to a liquid state, leading to the clear disappearance of wax crystals. This transformation has been effectively demonstrated using Scanning Electron Microscopy (SEM) (Yan et al., 2018; Yang et al., 2017a, 2017b). However, SEM has limitations in quantifying the texture and quality of surface waxes. Consequently, previous studies have primarily correlated changes in wax composition with greasiness scores rather than directly assessing wax morphology (Yan et al., 2018; Yang et al., 2017a, 2017b). A notable limitation of this approach is that greasiness scores are highly subjective and can vary between evaluators. To address this issue, Atomic Force Microscopy (AFM) was employed to objectively quantify the texture and quality of surface waxes using roughness parameters such as Rq, Ra, Rmax, and Rz.

Our results demonstrated a significant decrease in these roughness parameters with increasing greasiness levels and the disappearance of wax crystals. Among these parameters, Ra and Rq are the most commonly used roughness metrics (Kovalev et al., 2019; Wieland et al., 2001). Low values of Ra and Rq indicate a more consistent and smooth surface, and their decrease reflects a significant reduction in roughness on ‘Jonagold’ apples. Correlation analysis further revealed a significant negative correlation between all roughness parameters and the content of liquid ester compounds, indicating that the morphological changes in wax are due to the accumulation of liquid components. Moreover, there was no significant correlation between changes in solid components and roughness during the greasiness process, which may be related to the lack of significant changes in the content of solid wax components. Our results align with previous reports, strongly suggesting that the accumulation of liquid waxes is critical to the solid-liquid phase changes in greasy apples, as demonstrated by thermodynamic analysis (Yang et al., 2021) and random forest modeling (Lee et al., 2024).

In this study, we also found that the value of roughness parameters varies with different scanning areas. This result is related to the spatial wavelength or frequency dependence of roughness on the scanned area images (Boussu et al., 2005). For smaller scanning areas, only higher frequency roughness is included, whereas for larger scanning areas, both high-frequency and low-frequency roughness are included. Therefore, the roughness value is higher for larger scanning areas. Hence, when comparing the roughness of the apple surface with the progress of skin greasiness, it is meaningful to use results from the same scanning area for comparison. Compared to SEM, AFM offers higher resolution at the nanoscale and provides richer details in 3D images. Additionally, AFM samples do not require any pre-treatment, enabling continuous observation of fruit skins in a nearly living state, and the samples can still be used for SEM observation afterward (Zu et al., 2006). In summary, roughness parameters (Rq, Ra, Rmax, and Rz) obtained through AFM have significant applications for the quantitative assessment of the greasiness process. However, further research is needed to establish standardized evaluation criteria using these roughness parameters to predict the onset of greasiness.

### 4.3 Quantifying skin greasiness development via roughness parameters (Rq, Ra, Rmax and Rz) contributes to identifying key genes related to wax biosynthesis

The molecular biology of apple greasiness remains largely unexplored. Current research predominantly centers on the formation of linoleate esters (Jiang et al., 2022; Yan et al., 2022), which constitute the primary liquid ester component in greasy apples - a finding confirmed in this study. The fatty acid desaturation enzyme (FAD) is the rate-limiting enzyme in the synthesis of linoleic acid (Lee et al., 2020), crucial for desaturating oleic acid to linoleic acid and thereby providing a substrate essential for greasiness development. Transcriptome analysis of apples with varying susceptibilities to skin greasiness showed that *MdFAD2* expression increases during the development of skin greasiness in ‘Golden Delicious’ and ‘Granny Smith’ apples, but remains low in ‘Fuji’ apples, which are less prone to skin greasiness (Yan et al., 2022). Jiang et al. (2022) further demonstrated that *MdFAD2* promotes linoleate ester synthesis through homologous expression in apples and heterologous expression in tomatoes. Our findings support these conclusions, demonstrating that changes in roughness parameters Rq, Ra, and Rz are significantly positively correlated with *MdFAD2* expression. This indicates that *MdFAD2* plays a crucial role in the dissolution of wax crystals during the development of skin greasiness in ‘Jonagold’ apples. By linking wax morphology changes with gene expression through roughness parameters, we introduce a novel method for identifying key genes involved in apple greasiness development. This approach provides new insights and methodologies for exploring the mechanisms underlying apple greasiness. In addition, the genes *MdWSD1*, associated with ester synthesis, and *MdWBC11*, related to wax transport, were also found to be significantly positively correlated with Rq, Ra, and Rz. However, their specific functions remain unclear and warrant further investigation.

## 5 Conclusions

This study revealed significant morphological and chemical changes in the surface waxes of ‘Jonagold’ apples during the development of skin greasiness. Atomic Force Microscopy (AFM) analysis showed a marked reduction in wax roughness parameters (Ra, Rq, Rmax, Rz) as the wax crystals diminished. This reduction in roughness was inversely correlated with the accumulation of liquid wax esters. Additionally, the expression of key wax biosynthesis genes (*MdFAD2*, *MdWSD1*, and *MdWBC11*) was negatively correlated with Ra, Rq, and Rz. These findings suggest that liquid esters and these genes play crucial roles in the development of skin greasiness, consistent with prior studies on chemical, thermal, and structural changes. AFM proved invaluable for quantifying wax morphology through surface roughness measurements during the development of apple skin greasiness.

## Data Availability

The original contributions presented in the study are included in the article/**Supplementary Material**. Further inquiries can be directed to the corresponding authors.
