# Supplementary material for: Characterization of morphological and chemical changes using atomic force microscopy and metabolism assays: the relationship between surface wax and skin greasiness in apple fruit
Source: Front Plant Sci. 2024 Oct 16;15:1489005. doi: 10.3389/fpls.2024.1489005 (PMC11521941; doi:10.3389/fpls.2024.1489005)
Supplement: Supplementary file 1 [file Table1.docx]

**SUPPLEMENTARY TABLE 1 Wax compositions of the mature apple fruit at harvest in ‘Jonagold’**

| **Fomula** | **Content（mg m^-2^）** | **State (20 ℃)** | **RI** | **RI*** |
| --- | --- | --- | --- | --- |
| **Alkanes** | **4455±613** |  |  |  |
| Heneicosane (C_21_) | 16±5 | solid^a^ | 2099 | 2100 |
| Tricosane (C_23_) | 25±2 | solid^a^ | 2299 | 2300 |
| Pentadecane (C_25_) | 51±4 | solid^a^ | 2500 | 2500 |
| Hexacosane (C_26_) | 43±1 | solid^a^ | 2600 | 2600 |
| Heptacosane (C_27_) | 300±53 | solid^a^ | 2690 | 2700 |
| Octacosane (C_28_) | 83±11 | solid^a^ | 2800 | 2800 |
| Nonacosane (C_29_) | 3877±538 | solid^b^ | 2900 | 2900 |
| Triacontane (C_30_) | 60±11 | solid^b^ | 3000 | 3000 |
|  |  |  |  |  |
| **Fatty alcohols** | **2832±252** |  |  |  |
| Nonacosan-10-ol (C_29_-10) | 1953±96 | Solid^d^ | 2981 | u |
| Tetracosanol (C_24_) | 139±38 | solid^c^ | 2744 | 2741 |
| Hexacosanol (C_26_) | 292±56 | solid^c^ | 2941 | 2945 |
| Octacosanol (C_28_) | 293±41 | solid^c^ | 3138 | 3139 |
| Triacontanol (C_30_) | 155±26 | solid^b^ | 3334 | 3334 |
|  |  |  |  |  |
| **Fatty acids** | **383±39** |  |  |  |
| Palmitic acid (C_16_) | 74±1 | solid^b^ | 2048 | 2050 |
| Linoleic acid (C_18:2_) | 108±1 | liquid^b^ | 2215 | 2212 |
| Oleic acid (C_18:1_) | 88±12 | liquid^b^ | 2220 | 2222 |
| Stearic acid (C_18_) | 43±5 | solid^b^ | 2246 | 2246 |
| Lignoceric acid (C_24_) | 70±22 | solid^c^ | 2833 | 2838 |
|  |  |  |  |  |
| **Sesquiterpenoids** | **215±41** |  |  |  |
| α-farnesene | 166**±37** | Liquid^a^ | 1481 | 1508 |
| (E,E)-Farnesol | 49**±6** | Liquid^a^ | 1814 | 1800 |
|  |  |  |  |  |
| **Esters** | **346±71** |  |  |  |
| Propyl linoleate | -^f^ | Liquid^e^ | 2260 | 2284 |
| Propyl oleate | - | Liquid^e^ | 2265 | 2292 |
| Butyl linoleate | 142±40 | Liquid^e^ | 2356 | 2391 |
| Butyl oleate | - | Liquid^e^ | 2361 | 2383 |
| Pentyl linoleate | - | Liquid^e^ | 2419 | 2417 |
| Pentyl oleate | - | Liquid^e^ | 2424 | 2483 |
| Farnesyl ester | 204±91 | Liquid^e^ | 3170 | u |
|  |  |  |  |  |
| **Triterpenic acids** | **5568±475** |  |  |  |
| Ursonic acid | 4034±568 | solid | 3597 | u |
| Oleanolic acid | 1534±122 | solid | 3647 | u |

RI*: Retention index from NIST 17. u: unknown.^a^ The information was obtained from the product database system of Thermo Fisher Scientific (chemicals.thermofisher.cn). ^b^ The information was obtained from the product database system of Sigma-Aldrich (http://www.sigmaaldrich.com/). ^c^ The information was obtained from WINGCH Chemicals Database ([www.basechem.org).](http://www.basechem.org). d) ^[d](http://www.basechem.org). d)^ Ensikat et al. 2006. ^e^ the information was obtained from the authentic standards synthetized in our lab. ^f^ Not detected.
